# A Review of the Diagnostic Use of Fine-needle Aspiration Cytology for Tuberculosis Epididymo-orchitis: To Do or Not to Do

**DOI:** 10.7759/cureus.6532

**Published:** 2020-01-01

**Authors:** Ashish Sharma, Shivaraj Nagalli, Arun T Varughese, Arthur M Ayvazian

**Affiliations:** 1 Internal Medicine, Yuma Regional Medical Center, Yuma, USA; 2 Family Medicine, Yuma Regional Medical Center, Yuma, USA

**Keywords:** fnac, genitourinary tuberculosis, testicular neoplasm, testicular tumor marker, unilateral scrotal swelling, epididymo-orchitis

## Abstract

Isolated tuberculous epididymo-orchitis is a rare manifestation of the vast extrapulmonary tuberculosis (EPTB) disease spectrum, especially in developed nations, making it prone to delayed diagnosis or misdiagnosis and inadvertent orchiectomy. Several observational studies and case reports have been reported with the successful use of fine-needle aspiration cytology (FNAC) in diagnosing tuberculosis orchitis, thus avoiding inadvertent orchiectomy. Because tuberculous epididymo-orchitis can mimic testicular neoplasm, the use of FNAC is not prevalent in developed countries for fear of the seeding of tumor cells and there is a lack of consensus on the use of FNAC for diagnostic purposes in such patients.

We report a case of a 27-year-old man with an atypical presentation of genitourinary tuberculosis (TB) and its management. The case report also reviews the literature to discuss the available evidence and tries to answer the long-standing question on the role of FNAC in the diagnosis of tuberculous epididymo-orchitis. The currently available literature has demonstrated the safety and efficacy of FNAC in diagnosing TB epididymo-orchitis and, based on our review, the benefits of differentiating TB epididymo-orchitis from testicular malignancy using FNAC exceeds its minimal risk and must be considered to minimize clinical diagnosis error and unnecessary orchiectomy in low-risk patients.

## Introduction

Tuberculosis (TB) is a widely prevalent disease worldwide, especially in developing countries. The incidence of tuberculosis is decreasing worldwide. The US TB incidence in 2018 (2.8 per 100,000 persons) represented a 1.3% decrease from 2017; the rate among non-U.S.-born persons was >14 times that in US-born persons [1-2]. Patients who usually present with pulmonary tuberculosis with constitutional symptoms are comparatively easier to diagnose than patients with a rare presentation of extrapulmonary TB (EPTB) without any pulmonary or other constitutional symptoms. Tuberculous epididymo-orchitis can present as isolated scrotal swelling without any classic constitutional symptoms and signs, particularly in patients with no prior history of TB, which then can pose a diagnostic and treatment challenge. FNAC is usually not done because of high suspicion for the neoplastic process based on imaging studies and a fear of the seeding of tumor cells. This may lead to inadvertent orchiectomy in such patients. This case highlights this diagnostic dilemma, along with some challenges of immigrant refugee health, and serves as a clinical reminder to keep a high degree of suspicion for prevalent epidemiological diseases when caring for patients of developing nations. The aim of this case presentation with a review of literature is to emphasize the importance of considering this rare presentation of tuberculosis high in the differential diagnosis to avoid delayed diagnosis or misdiagnosis, especially in patients with risk factors of TB exposure, immunocompromised disease, and patients from developing countries. Fine needle aspiration cytology (FNAC) in the absence of testicular tumor markers should be considered to avoid inadvertent orchiectomy.

## Case presentation

A 27-year-old Hispanic male from Guatemala, with no past medical and family history, was brought to the hospital by the border patrol for the complaint of right-sided scrotal pain and gradually increasing swelling started about one month before presentation. The patient denied any abdominal pain, dysuria, sexually transmitted disease, cough, weight loss, night sweats, or any other symptoms. On arrival, he was febrile with temperature 101.9 F, with other vital signs being unremarkable. Only significant findings on the physical exam were marked swelling and induration, with mild tenderness of the right testis and epididymis. Lab workup included a normal complete blood count and normal renal function. Urinalysis was unremarkable with negative chlamydia and gonorrhea urine polymerase chain reaction (PCR).

**Figure 1:**
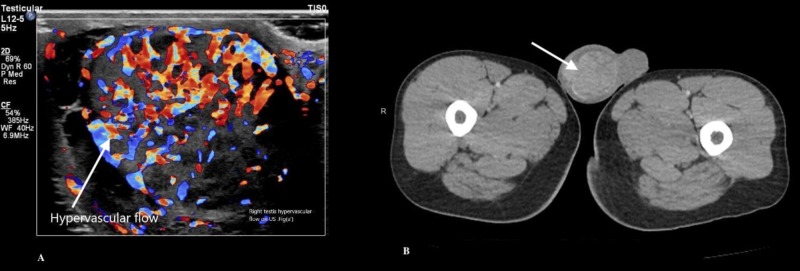
Testicular US Doppler (A) and CT scan of pelvis (B) showing heterogenous texture, multiple hypoechoic masses, with septations and hypervascular flow in the right testis US: ultrasound; CT: computed tomography

A testicular ultrasound showed an irregular heterogeneous echotexture of the right testicle with multiple hypoechoic masses with septation, microcalcification, and increased hypervascular flow, as shown in Figure 1A. The left testicle was normal in echotexture and morphology without any solid or cystic intratesticular masses. The patient was started on antibiotics for bacterial right epididymitis-orchitis. He continued to have fever despite intravenous (IV) antibiotics. A chest X-ray did not show any acute cardiopulmonary findings. Urine and blood culture remained negative. A computed tomography (CT) scan of the abdomen and pelvis with contrast showed a heterogeneously enhancing, enlarged right testicle (as shown in Figure 1B), epididymis, seminal vesicle, as well as retroperitoneal and mesenteric lymphadenopathy.

CT scan of the chest with contrast showed pleural thickening with bilateral pleural effusion, compressive atelectasis, and multiple bilateral mildly enlarged hilar lymph nodes. Further investigation with tumor markers, including alpha-fetoprotein (AFP), beta-human chorionic gonadotropin (hCG), and lactate dehydrogenase (LDH), was unremarkable. Human immunodeficiency virus (HIV) was also ruled out. Three successive sputum samples for consecutive three days were negative for acid-fast bacilli (AFB) stain. For the findings of the CT scans showing lymphadenopathy, a right indurated testicular mass, and poor response to antibiotics, a right radical orchiectomy was done for a suspected diagnosis of right testicular neoplasm with secondary infection. Biopsy and FNAC of the testis were not done to avoid causing the seeding of tumor cells to the inguinal lymph nodes because of suspicion of the presence of a cancerous right testicular mass.

**Figure 2:**
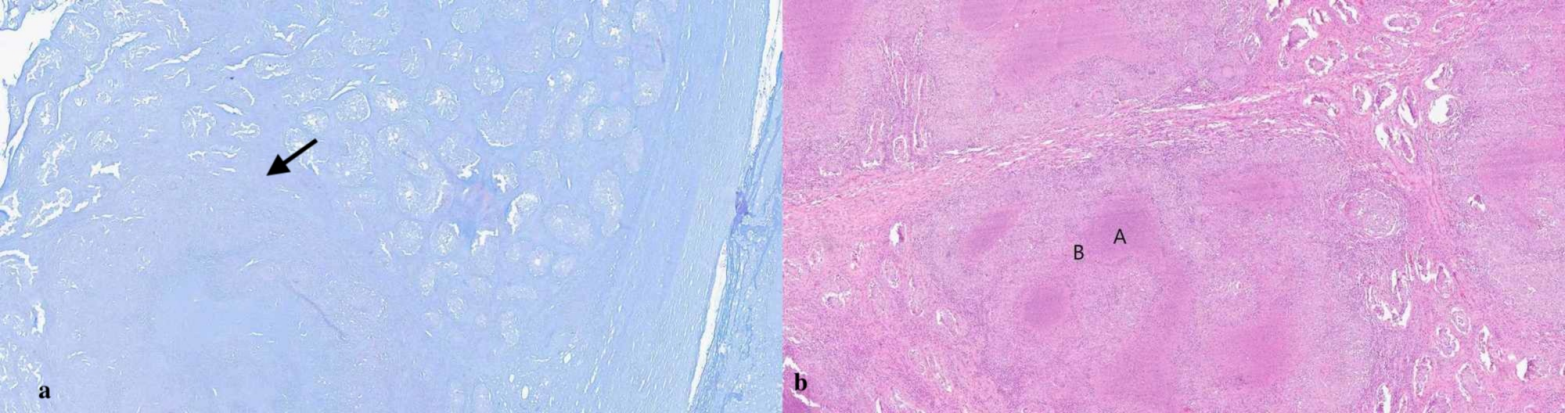
AFB slide of the right testis (a) showing AFB as red dots. Hematoxylin and eosin slide (b) showing caseous necrosis (A) and peripheral lymphocytes infiltration (B) AFB: acid-fast bacilli

Testicular tissue histopathological examination and AFB stain showed caseating granulomas and acid-fast bacilli, as shown in Figure 2. Treatment with isoniazid (INH), rifampin, pyrazinamide, ethambutol therapy, along with vitamin B6 initiated. Bronchoscopy was done post-orchiectomy, which revealed a negative AFB stain but positive Mycobacterium tuberculosis PCR. AFB culture from the sputum sample and bronchoalveolar lavage were positive for after one week. The patient's fever resolved after the initiation of anti-tuberculous therapy. The patient was discharged to the border patrol, with the recommendation to continue following up with the public health department for ongoing anti-tuberculous therapy. The last known disposition of the patient was in the nearby detention center. Outpatient follow-up with the public health department showed a resolution of the patient's symptoms.

## Discussion

Urogenital TB is the third most common presentation of EPTB after lymph node and pleural effusion, accounting for 2% to 20% of total cases of TB epididymo-orchitis accounts for 10%-50% of the total cases of EPTB [1]. Tuberculous epididymo-orchitis can present as painful or painless swelling. Fever and other constitutional symptoms of TB, such as weight loss, cough, and night sweats, can be present or absent. Other clinical manifestations include scrotal or testicular nodularity, prostate nodularity, non-healing ulcer, fistula of genitalia, and groin lymphadenopathy. Epidemiological risk factors include a prior history of TB or exposure, traveling to or being a past or present resident of a TB endemic area [3].

The differential diagnosis in patients with testicular epididymo-orchitis can be broad. Symptoms localized to the single scrotum can be present in testicular neoplasm, bacterial epididymo-orchitis, genitourinary sarcoidosis, and hernia with hydrocele. Many times, an atypical presentation can cause either delayed diagnosis or misdiagnosis and can lead to orchiectomy, as it mimics testicular tumors. Diagnostic tools to diagnose genitourinary TB include urine acid-fast stain, mycobacterial culture, and polymerase chain reaction (PCR) for Mycobacterium tuberculosis and radiographic studies [4-5]. Positive urine AFB stain is non-diagnostic, as non-Mycobacterium tuberculosis AFB can be positive as well [5]. Pulmonary TB also should be evaluated with a sputum AFB stain and culture or bronchoscopy if sputum cannot be obtained to confirm the diagnosis [6]. Treatment for EPTB follows a similar recommendation as pulmonary TB with isoniazid (INH), rifampin, pyrazinamide, ethambutol, and B6 for six months. Complications of delayed diagnosis or misdiagnosis of tuberculosis can cause significant damage to the patient’s health. Some of the most severe complications include urogenital destruction, renal failure, non-healing wound, ulcer with abscess, and infertility, among many others.

In our case, the patient’s presentation with only fever and unilateral scrotal swelling prompted a diagnosis of the more common bacterial epididymo-orchitis. After an inadequate response to appropriate antibiotic therapy, further imaging studies were obtained. For findings of lymphadenopathy and pleural effusion on imaging, the patient also underwent bronchoscopy and sputum AFB stain, which was negative, and cultures only became positive after one week. Right orchiectomy was carried out for concern of underlying testicular malignancy. Although the tumor markers were negative in our case, studies have shown that 25% of seminoma testicular neoplasm can have beta-hCG elevation without AFP elevation in contrast to non-seminomatous germ cell tumor where beta-hCG and AFP can be elevated in 80%-85% cases [7-8].

Many times, an atypical presentation can cause either delayed diagnosis or misdiagnosis and can lead to inadvertent orchiectomy, as it mimics testicular tumors. Kinnear et al. reported a case where the patient with a similar presentation initially diagnosed with bacterial epididymo-orchitis and treated with antibiotics first [9]. Later, the patient represented with a testicular abscess formation, which was confirmed with incision and drainage, and the patient was then started on anti-tuberculous therapy. In our case, the patient remained an inpatient until the final diagnosis because of persistent symptoms of fever, testicular swelling, and inability to access outpatient follow-up medical care. He never developed any ulcer, fistula, or clinically appreciable abscess on daily physical examination. In the literature, multiple similar and atypical presentations of EPTB have been reported after being misdiagnosed or delayed diagnosis with the patient eventually getting inadvertent orchiectomy for similar reasons [9-13].

Handa et al. and Gupta et al., in their retrospective analysis of a combined more than 250 patients, found FNAC useful in 90% cases for a diagnosis of tuberculous orchitis [14-15]. Viswaroop et al. reported a case series of 40 patients with epididymal tuberculosis in which 26 patients diagnosed with FNAC, nine patients required tissue biopsy, and five patients underwent orchiectomy for the suspicion of a testicular tumor and diagnosed with histopathology specimen [16]. There are many case reports and case series in the literature on diagnosing tuberculous orchitis with FNAC and epididymal nodule biopsy [17-18]. It has been observed that the theoretical risk of the seeding of cancer to skin and lymph nodes with FNAC usually precludes its use; however, the seeding of tumor cells with FNAC remains unproven. Shah et al., in his research, reported a 95% non-neoplastic lesion in their study of epididymis nodules with FNAC [19]. In our reviews of the literature, most of these cases are reported from developing nations where clinical suspicion for tuberculous orchitis remains high, given the high tuberculosis prevalence in these areas.

Nonetheless, in our opinion, based on a literature review, FNAC Is a cost-effective and less invasive procedure. It is easy to perform and has a low risk of adverse effects and complications. The diagnostic accuracy of FNAC is increased when a cytopathologist is involved in the interpretation of the smear cytology. FNAC is also very quick, and the results of the smear can be obtained rapidly to guide surgical necessity by the urologist. It can serve as an essential tool in the diagnosis of a suspected case of tuberculous epididymo-orchitis, with the benefit of avoiding inadvertent orchidectomy. FNAC should be considered if epidemiologic risk factors for TB are present and especially if tumor markers are negative.

## Conclusions

Our case report illustrates the diagnostic challenges associated with an atypical and rare presentation of the EPTB. The condition has been long-recognized and well-illustrated in the medical literature. However, it remains mostly unknown, especially to physicians trained in developed nations given the low prevalence of tuberculosis, causing delayed diagnosis and putting patients to grave risks of further complications. It also highlights the challenge of immigrant refugee health where a diagnosis delay or misdiagnosis may lead to catastrophic results because of poor access and follow up to appropriate health care services. It serves as a learning lesson for keeping a high suspicion for the patients with risk factors and the appropriate use of FNAC in a patient with less suspicion for an underlying neoplasm in the absence of elevated tumor markers that can minimize clinical diagnosis error and unnecessary orchiectomy. Currently, available literature has demonstrated the safety and efficacy of FNAC in diagnosing TB epididymo-orchitis. We believe that the risk of the spread of tumor cells is overestimated. Hence, the benefits of differentiating TB epididymo-orchitis from testicular malignancy using FNAC exceeds its minimal risk. Further clinical studies focused on the diagnostic yield of FNAC and complication rates like seeding of tumor cells can provide more clarity and establish it as a standard of care.
